# Inventory and DNA-barcode library of ground-dwelling predatory arthropods from Krokar virgin forest, Slovenia

**DOI:** 10.3897/BDJ.10.e77661

**Published:** 2022-03-09

**Authors:** Žan Kuralt, Urška Ratajc, Neža Pajek Arambašić, Maja Ferle, Matic Gabor, Ivan Kos

**Affiliations:** 1 University of Ljubljana, Biotechnical Faculty, Department of Biology, Ljubljana, Slovenia University of Ljubljana, Biotechnical Faculty, Department of Biology Ljubljana Slovenia; 2 National Institute of Biology, Ljubljana, Slovenia National Institute of Biology Ljubljana Slovenia

**Keywords:** Araneae, chilopoda, geophilomorpha, scolopendromorpha, lithobiomorpha, coleoptera, Carabidae, faunistics, primary forest

## Abstract

**Background:**

At a time of immense human pressure on nature and the resulting global environmental changes, the inventory of biota - especially of undisturbed natural areas - is of unprecedented value as it provides a baseline for future research. Krokar, an example of such an undisturbed area, is the largest virgin forest remnant in Slovenia. It is located in the Dinaric Alps, which are believed to harbour the most diverse fauna of soil invertebrates in Europe. Nevertheless, the soil fauna of the Krokar virgin forest has not been thoroughly studied. Moreover, modern taxonomic approaches often rely on genetic information (e.g. DNA-barcodes), while extensive reference libraries from the Dinaric area are lacking. Our work, therefore, focused on addressing this lack of faunistic and genetic data from the Dinaric area.

**New information:**

A total of 2336 specimens belonging to 100 taxa (45 spiders, 30 centipedes, 25 ground-dwelling beetles) were collected and deposited to GBIF. DNA-barcodes of 124 specimens belonging to 73 species were successfully obtained and deposited in GenBank and BOLD databases.

## Introduction

The European landscape is probably one of the most fragmented on the planet. Forests that once covered vast areas have undergone significant changes in the past and now exist only in relatively small fragments (Estreguil et al. 2013). Amongst them, the proportion of primary forests is vanishingly small, accounting for 0.7% of Europe’s forest area (Sabatini et al. 2018). However, these forests are essential forest ecosystems that encompass all stages of forest development. They also provide habitat for a large number of fungi, plants and animals and serve as an extensive scientific resource (Navarro and Pereira 2012). Primary forests preserve natural ecological processes and are, therefore, resilient to natural disturbances (Thompson et al. 2009, Král et al. 2014).

European primary forests are mainly located in boreal and alpine regions (Sabatini et al. 2018). The virgin forest remnant Krokar (hereafter Krokar) is an example of the latter. It is located on the Borovec Mountain in southern Slovenia, in the Dinaric Mountains, which extend for 650 km from NW to SE and form an orographic barrier between the Adriatic Sea and the Pannonian Basin (Mihevc et al. 2010). The area served as a glacial refugium during the Pleistocene (Hewitt 2000, Brus 2010, Simaiakis and Strona 2015), its diverse landscape and relatively mild climate with high precipitation allowing for a diverse flora and fauna with high endemism (Griffiths et al. 2004).

In the face of climate change, however, the Dinaric Mountains are likely to be as vulnerable as other mountain regions of the world (Beniston 2003). The effects of global change on alpine ecosystems have been observed many times, affecting environmental morphology, vegetation and soils. Several studies have reported upward shifts in vegetation (up to 4 m per decade) and increased erosion (Pauli et al. 1996, Theurillat and Guisan 2001, Nearing et al. 2004, Gehrig‐Fasel et al. 2007, Rounsevell and Loveland 2013, Chersich et al. 2015, Robinson et al. 2018). In addition, Pizzolotto et al. (2014) reported similar findings for Carabid beetles in the Dolomites. Knowledge of the current status of plant and animal communities is, therefore, of great importance and allows the assessment of changing climate and human impact (Tuf and Tufova 2008, Bauhus et al. 2009, Cluzeau et al. 2012, Burrascano et al. 2013, Bončina et al. 2017).

Whilst the structure and forest development of Krokar have been thoroughly studied (Diaci 2002, Kraigher et al. 2002, Kutnar et al. 2002, Piltaver et al. 2002, Diaci et al. 2008, Grce 2010, Bončina 2011, Nagel et al. 2012, Kamenik 2013), the diversity of ground-dwelling invertebrates is largely unknown. Nevertheless, some studies have already found a high diversity of predatory invertebrates, such as centipedes (Kos 1996, Griffiths et al. 2004, Grgič and Kos 2005, Ravnjak and Kos 2015, Simaiakis and Strona 2015, Bonato et al. 2017a, Peretti and Bonato 2018) in the Dinarics. Ground-dwelling invertebrates play an important role in forest soil processes (e.g. nutrient cycling, pedogenesis). Predators (e.g. spiders, centipedes and certain groups of beetles) play an important role in regulation and, thus, indirectly influence these processes (Lavelle et al. 2006). They respond rapidly to habitat changes and, because of their position as mesopredators in the trophic cascade, are also highly sensitive to changes at lower trophic levels (Maelfait 1996, Paoletti et al. 1996, Rainio and Niemelä 2003, Pearce and Venier 2006, Koivula 2011, Schreiner et al. 2012, Gerlach et al. 2013).

The main objectives of the study were: (1) to generate a checklist of soil and ground-dwelling predatory arthropods in the study area and (2) to build a DNA-barcode library of these taxa.

## Sampling methods

### Study extent

Krokar is located on Mount Borovec in the Dinaric Mountains in southern Slovenia (45.540333°N, 14.764737°E) and covers an area of 74.5 hectares at an altitude of 880 to 1190 m a.s.l. The dolomite bedrock of the northern part is gradually replaced by limestone towards the south, resulting in a diverse and rugged terrain. The average annual temperature is 5°C with 2000 mm of precipitation (Grce 2010). The predominant forest communities are Omphalodo-Fagetum, Isopryo-Fagetum and Orvalo-Fagetum (Bončina and Robič 1993). Krokar was excluded from management plans in 1885 (Hočevar et al. 1985) and declared a special purpose forest in 2005 under the Regulation of protective forests and forests with special purpose (Uradni list RS, št. 88/05, 56/07, 29/09, 91/10, 1/13 in 39/15 2005). Finally, it was declared a UNESCO natural heritage area in 2017 (UNESCO 2017).

**Figure 1.**
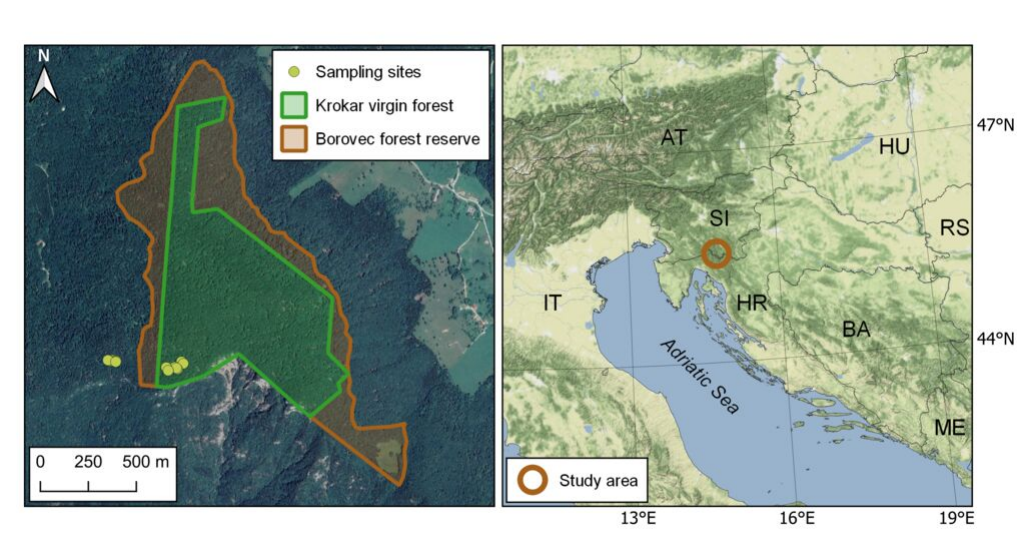
Map on the left shows Borovec Forest Reserve and Krokar virgin forest where sampling was performed (Map data ©2015 Google). Map on the right displays a wider area of the study site location (Map tiles by Stamen Design, under CC BY 3.0. Data by OpenStreetMap, under ODbL).

Parallel sampling was conducted in an adjacent secondary forest (45.53891°N, 14.76478°E), located approximately 300 m west of the sampling sites in Krokar (see Figure 1), with similar geographic, geologic and climatic characteristics. The sampling sites there were located in sloping terrain with varying stages of forest development.

### Sampling description

**Collecting methods**

We used a variety of non-selective sampling methods to minimise collector bias. The selected methods also allowed for efficient collection of both endogeic and ground-dwelling species (Bonato et al. 2017). Two sets of five pitfall traps were set in patches with different forest developmental stages (sapling, pole and sawlog). Similarly, six soil samples per developmental stage were collected.

Soil samples were collected approximately 15 cm deep in the soil using a soil corer with a diameter of 21 cm. Litter and fermentative layers were also collected. Macroinvertebrates were later extracted for one month using modified Tullgren funnels with a cooled funnel base and ethylene glycol as a preservative. The extracted animals were then sorted, identified and preserved in 96% ethanol at -20°C for molecular methods.

Leaf litter was sampled using a sieve with a diameter of 38 cm and a mesh size of 13×13 mm over a white cloth. They were then collected with an aspirator and forceps and preserved in 96% ethanol and later stored at -20°C.

Pitfall traps were set using white plastic cups with a diameter of 10 cm and transparent plastic rain cover, filled with ethylene glycol and set in a line of five traps 1 m apart. After 7–10 days, the contents of the traps were collected, sorted, preserved in 96% ethanol and stored at -20°C.

**Specimen identification**

Spider and centipede specimens were observed using an Olympus SZX7 stereomicroscope, while beetles were observed using an Olympus SZ61 stereomicroscope. Smaller centipedes were mounted on permanent microscopic slides and observed with an Olympus CX41 microscope.

Adult spiders were identified using standard identification keys (Roberts 1995, Nentwig et al. 2020, Oger 2020). If the morphology of the female epigyne was not discernible, the epigyne was dissected and macerated overnight in 15% potassium hydroxide (KOH) to remove soft tissue. For taxonomy and nomenclature, we followed the World Spider Catalog (World Spider Catalog 2021).

Centipedes were identified according toMatic (1966), Matic (1972), Koren (1986), Koren (1992), Stoev et al. (2010) for Lithobiomorpha; Brölemann (1930) and Lewis (2011) for Scolopendromorpha; ChiloKey (Bonato et al. 2014) for Geophilomorpha. For taxonomy and nomenclature, we followed ChiloBase 2.0 (Bonato et al. 2016).

Beetles were identified using the determination keys from “Die Käfer Mitteleuropas" by Freude et al. (1974) and the subsequent editions.

**DNA extraction and sequencing**

Genomic DNA was isolated from one of the legs or the whole animal (depending on the size of the specimen). DNA extraction was performed with the MagMAX DNA Multi-sample Kit (Thermo Fisher Scientific Inc., United States) used on a Microlab STAR (Hamilton, United States) pipetting robot. We used the KAPA2G Robust PCR Kit (Sigma-Aldrich, United States) to amplify the mitochondrial cytochrome oxidase I (COI) gene. A 650 bp long fragment of COI was amplified using primers LCO1490 and HCO2198 (Folmer et al. 1994). PCR began with initial denaturation for 3 min at 95°C, followed by 35 cycles of denaturation (30 sec at 95°C), annealing (30 sec at 48°C), elongation (60 sec at 72°C) and then final elongation for 3 min at 72°C. PCR products were purified with Exonuclease I and FastAP (Thermo Fisher Scientific Inc., United States) according to the manufacturer’s instructions. Each fragment was sequenced in both directions using PCR amplification primers from Macrogen Europe (Amsterdam, The Netherlands).

Using Geneious Prime software (Biomatters, New Zealand), we assembled forward and reverse reads, trimmed and manually inspected for possible base-calling errors. Finally, we translated the sequences using all six reading frame positions to ensure that no stop codons were present and generated consensus sequences. For verification, we performed BLAST searches to confirm the identity of all new sequences as either centipede, spider or ground-dwelling beetle barcodes, based on previously-published sequences (high identity values, very low E-values).

In order to investigate the relations amongst the DNA-barcoded taxa, we built a COI tree using Geneious Prime Tree Builder (Geneious version 2022.0 created by Biomatters). Distance matrix was calculated using Global alignment with free end gaps and 70% similarity (IUB)(5.0/-4.5) cost matrix, while the tree was built with Tamura-Nei genetic distance and the Neighbour-Joining tree build method.

## Geographic coverage

### Description

The study area includes Krokar virgin forest (74.49 ha) and an adjacent secondary forest. Both sites are situated on Borovec Mountain in the northern Dinaric Alps (Fig. 1).

### Coordinates

45.53630 and 45.55152 Latitude; 14.76796 and 14.78080 Longitude.

## Taxonomic coverage

### Description

The database contains data on 2336 specimens we collected and identified (1079 spiders, 323 ground-dwelling beetles, 299 geophilomorphs, 386 lithobiomorphs, 249 scolopendromorphs). See Suppl. material 1 for list of specimens. The dataset was deposited to GBIF (https://doi.org/10.15468/72ytmh).

### Taxa included

**Table:** 

Rank	Scientific Name	Common Name
order	Araneae	spider
class	Chilopoda	centipedes
order	Coleoptera	beetles

## Temporal coverage

### Notes

Collecting was conducted between October 2018 and August 2019 (see Table 1).

## Collection data

### Collection name

Ground-dwelling invertebrates of Krokar virgin forest.

### Collection identifier

KROK-1819

### Parent collection identifier

KROK

### Specimen preservation method

96% ethanol, some smaller centipedes are mounted on microscopic slides.

## Usage licence

### Usage licence

Creative Commons Public Domain Waiver (CC-Zero)

## Data resources

### Data package title

Soil and ground-dwelling predatory arthropods (Araneae; Chilopoda: Geophilomorpha, Lithobiomorpha, Scolopendromorpha; Coleoptera: Carabidae, Staphylinidae) of Borovec Mountain and Krokar virgin forest.

### Number of data sets

2

### Data set 1.

#### Data set name

Soil and ground-dwelling predatory arthropods (Araneae, Chilopoda, Carabidae) of Borovec Mountain and Krokar virgin forest.

#### Number of columns

13

#### Description

List of all collected and identified specimens. GenBank accession codes and BOLD process IDs of DNA-barcoded specimens are listed in the *GenBankAccession* and *boldSequenceID* columns.

**Data set 1.** 

Column label	Column description
eventID	An identifier of the sampling event, corresponding to the eventID in the "Sampling events" dataset.
order	The name of the order.
scientificName	The full scientific name, with authorship and date information, if known.
sex	The sex of the specimen, if applicable.
taxonRank	The taxonomic rank of the most specific name in the scientificName.
identifiedBy	A list (concatenated and separated) of names of people, groups or organisations who assigned the Taxon to the subject.
dateIdentified	The date on which the subject was identified as representing the Taxon.
basisOfRecord	The specific nature of the data record.
preparations	Type of preservative. Either AP (alcohol preparation) or MP (microscopic slide preparation)
GenBankAccession	GenBank accession code.
occurrenceID	Unique occurrence identifier.
lifeStage	Life stage of specimen. Either adult, subadult or juvenile.
boldSequenceID	Sequence identifier at boldsystems.com

### Data set 2.

#### Data set name

Sampling events

#### Number of columns

11

**Data set 2.** 

Column label	Column description
eventID	An identifier for the sampling event.
eventDate	Date of sampling event.
geodeticDatum	Coordinate reference system of coordinates.
habitat	Forest type, either virgin forest or secondary forest and forest development stage, either sapling, pole or sawlog.
decimalLatitude	The geographic latitude (in decimal degrees, using the WGS84 spatial reference system).
decimalLongitude	The geographic longitude (in decimal degrees, using the WGS84 spatial reference system).
minimumElevationInMetres	Elevation of the sampling site.
samplingMethod	The name of the sampling method used in sample collection.
coordinateUncertaintyInMetres	Uncertainty of coordinates in metres.
recordedBy	A list of names of people responsible for collecting of samples.
country	The name of the country in which the location occurs.

## Additional information

### Summarized results

The taxonomical structure of the dataset is represented by 100 different species - 72 species from Krokar, 80 from the secondary forest and 52 species from both sites. A total of 30 centipede species, 45 spider species and 25 ground-dwelling beetle species are included in the dataset. The most abundant centipede species were *Lithobiuspygmaeus* (225 specimens), *Cryptopshortensis* (129), *Strigamiaacuminata* (116) and *Cryptopsparisi* (103) and, for spiders, *Inermocoelotesinermis* (202), *Harpactealepida* (172), *Histoponaluxurians* (154), *Micronetaviaria* (133) and *Comaromasimoni* (105) and, amongst ground-dwelling beetles, *Aptinusbombarda* (125), followed by *Pterostichusburmeisteri* (71). DNA-barcoded specimens are listed in Table 2.

We collected an old-growth forest specialist *Carabusirregularis* and some Balkan/Dinaric endemics, namely *Carabuscaelatus*, *Carabuscroaticus*, *Dysderaadriatica*, *Amaurobiusobustus*, *Histoponaluxurians* and *Centrophantesroeweri*, *Harpolithobiusgotcheensis*, *Lithobiusanici* sp.n., *Lithobiuscarniolensis* and *Cryptopsrucneri*.

A few of the spider species are considered rare according to the Spiders of Europe (Nentwig et al. 2020). These include *Amaurobiusobustus* (rare), *Coelotesatropos* (rarely found), *Scotarguspilosus* (very rarely found) and *Walckenaeriasimplex* (very rarely found). The finding of *Erigoneautumnalis* and *Mermessustrilobatus*, both spiders of North American origin, in this remote area, indicates their alarming invasive potential and suggests a wider distribution than known or expected. Their impact on native (spider) fauna is also unknown and should be studied in the future.

The specimens identified as Lithobius (Sigibus) anici sp.n. belong to an undescribed species that has already been recorded at various localities in the Dinaric parts of Slovenia and Bosnia and Herzegovina. Its currently known area of distribution suggests that the species is endemic to the Dinarics, although further studies are needed to confirm this claim.

Comprehensive voucher information, taxonomic classifications, DNA barcode sequences and trace files (including their quality) are publicly accessible through the public dataset “DS-KROK4BDJ” (Dataset ID: dx.doi.org/10.5883/DS-KROK4BDJ) on the Barcode of Life Data Systems (BOLD; www.boldsystems.org) (Ratnasingham and Hebert 2007). In addition, all new barcode data were deposited in GenBank.

**Figure 2.**
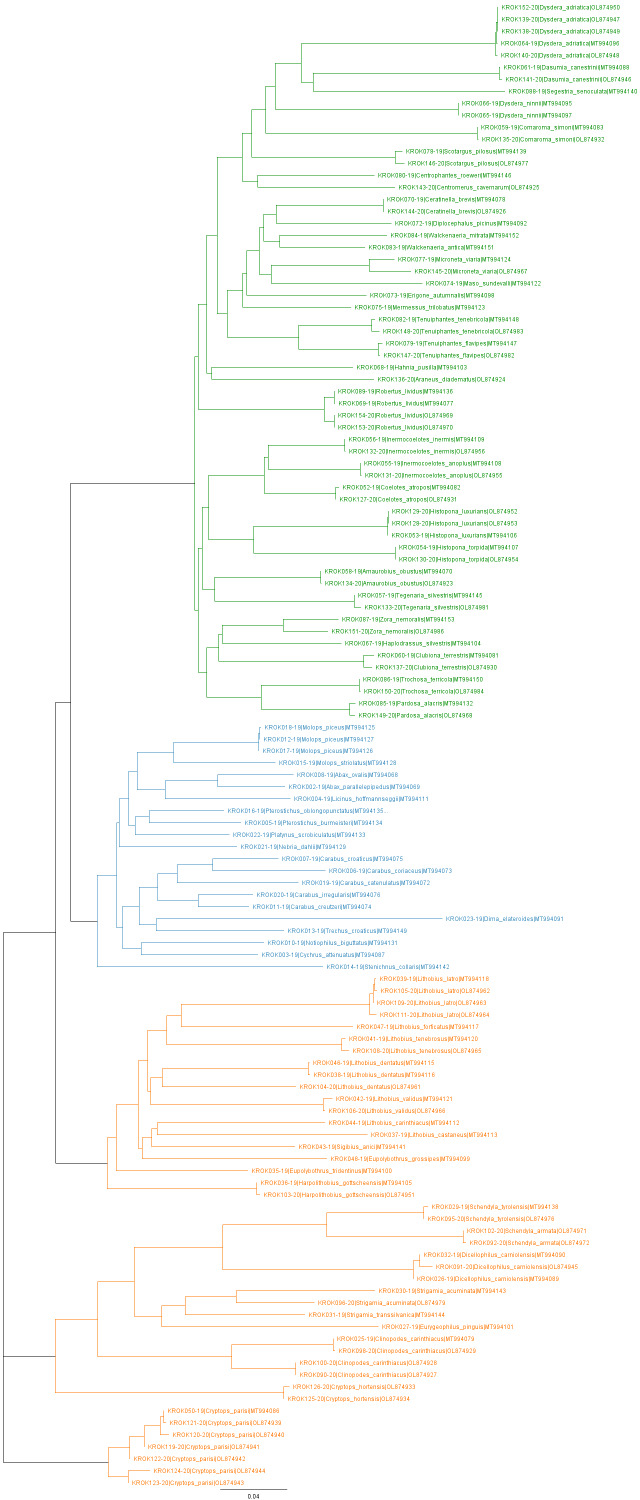
COI tree of DNA-barcoded taxa. Tree branches and labels are coloured according to the predator group (green for spiders, blue for ground-dwelling beetles, orange for centipedes). The tree was constructed in Geneious Prime (Geneious version 2022.0 created by Biomatters).

The COI tree (Fig. 2) of DNA-barcoded taxa is showing a topology consistent with the current knowledge of relationships between the taxa included. There are, however, a few species with deep genetic differences, that could be explained by the fact that the area served as a glacial refugium during the Pleistocene, which resulted in high intraspecific genetic diversity or even cryptic species. For instance, two DNA-barcoded specimens of *Zoranemoralis* show deep genetic difference, although they were identified as such, based on genital and palpal morphology. Similarly, there is a deep genetic difference between two specimens of *Strigamiaacuminata*. The specimens were placed into separate unique BINs - BOLD:AEB5728 and BOLD:AEG5654 with distances (p-dist) to nearest neighbour being 7.85% and 10.42%, respectively. Since the divergence of Western and Eastern Alps populations of *S.acuminata* was estimated to around 14 Ma (Bonato et al. 2017b), we could presume that the turbulent events of Neogene and Quaternary - especially Pleistocene - could lead to the observed cryptic diversity.

## Supplementary Material

EC9DDDA3-15A9-5AB6-A067-834D370F6780 10.3897/BDJ.10.e77661.suppl1 Supplementary material 1 Specimen list Data type dataset Brief description List of specimens collected during field excursions to Mount Borovec and Krokar virgin forest. File: oo_640399.csv https://binary.pensoft.net/file/640399 Žan Kuralt, Urška Ratajc, Neža Pajek Arambašić, Maja Ferle, Matic Gabor, Ivan Kos

58655C74-7D59-5703-8F04-2A3B992BD430 10.3897/BDJ.10.e77661.suppl2 Supplementary material 2 Sampling events Data type dataset Brief description Field excursions to Mount Borovec and Krokar virgin forest. File: oo_637986.tsv https://binary.pensoft.net/file/637986 Žan Kuralt, Urška Ratajc, Neža Pajek Arambašić, Maja Ferle, Matic Gabor, Ivan Kos

## Figures and Tables

**Table 1.** List of field excursions to Borovec Mountain. See Suppl. material 2 for full list of sampling events.

**Date**	**Locality**	**Sampling method**	**No. of soil cores / pitfall traps**
17.10.2018	Krokar, secondary forest	leaf litter sifting	
17.10.2018	Krokar, secondary forest	soil sampling	36
17.10.–25.10.2018	Krokar	pitfall traps	30
4.1.2019	Krokar, secondary forest	soil sampling	36
4.1.2019–16.1.2019	Krokar, secondary forest	pitfall traps	36
19.4.2019–7.5.2019	Krokar, secondary forest	pitfall traps	60
17.5.2019–28.5.2019	Krokar, secondary forest	pitfall traps	60
1.8.2019–9.8.2019	Krokar, secondary forest	pitfall traps	60

**Table 2.** DNA-barcoded specimens with GenBank accession codes and BOLD process IDs.

order	scientificName	GenBankAccession	boldSequenceID
Araneae	*Amaurobiusobustus* L. Koch, 1868	OL874923	KROK134-20
Araneae	*Amaurobiusobustus* L. Koch, 1868	MT994070	KROK058-19
Araneae	*Araneusdiadematus* Clerck, 1757	OL874924	KROK136-20
Araneae	*Centromeruscavernarum* (L. Koch, 1872)	MT994077	KROK069-19
Araneae	*Centromeruscavernarum* (L. Koch, 1872)	OL874925	KROK143-20
Araneae	*Centrophantesroeweri* (Wiehle, 1961)	MT994146	KROK080-19
Araneae	*Ceratinellabrevis* (Wider, 1834)	OL874926	KROK144-20
Araneae	*Ceratinellabrevis* (Wider, 1834)	MT994078	KROK070-19
Araneae	*Clubionaterrestris* Westring, 1851	MT994081	KROK060-19
Araneae	*Clubionaterrestris* Westring, 1851	OL874930	KROK137-20
Araneae	*Coelotesatropos* (Walckenaer, 1830)	MT994082	KROK052-19
Araneae	*Coelotesatropos* (Walckenaer, 1830)	OL874931	KROK127-20
Araneae	*Comaromasimoni* Bertkau, 1889	MT994083	KROK059-19
Araneae	*Comaromasimoni* Bertkau, 1889	OL874932	KROK135-20
Araneae	*Dasumiacanestrinii* (L. Koch, 1876)	MT994088	KROK061-19
Araneae	*Dasumiacanestrinii* (L. Koch, 1876)	OL874946	KROK141-20
Araneae	*Diplocephaluspicinus* (Blackwall, 1841)	MT994092	KROK072-19
Araneae	*Dysderaadriatica* Kulczynski, 1897	OL874949	KROK138-20
Araneae	*Dysderaadriatica* Kulczynski, 1897	OL874947	KROK139-20
Araneae	*Dysderaadriatica* Kulczynski, 1897	MT994096	KROK064-19
Araneae	*Dysderaadriatica* Kulczynski, 1897	OL874948	KROK140-20
Araneae	*Dysderaadriatica* Kulczynski, 1897	OL874950	KROK152-20
Araneae	*Dysderaninnii* Canestrini, 1868	MT994097	KROK065-19
Araneae	*Dysderaninnii* Canestrini, 1868	MT994095	KROK066-19
Araneae	*Erigoneautumnalis* Emerton, 1882	MT994098	KROK073-19
Araneae	*Hahniapusilla* C. L. Koch, 1841	MT994103	KROK068-19
Araneae	*Haplodrassussilvestris* (Blackwall, 1833)	MT994104	KROK067-19
Araneae	*Histoponaluxurians* (Kulczynski, 1897)	MT994106	KROK053-19
Araneae	*Histoponaluxurians* (Kulczynski, 1897)	OL874953	KROK128-20
Araneae	*Histoponaluxurians* (Kulczynski, 1897)	OL874952	KROK129-20
Araneae	*Histoponatorpida* (C.L.Koch, 1837)	MT994107	KROK054-19
Araneae	*Histoponatorpida* (C.L.Koch, 1837)	OL874954	KROK130-20
Araneae	*Inermocoelotesanoplus* (Kulczynski, 1897)	OL874955	KROK131-20
Araneae	*Inermocoelotesanoplus* (Kulczynski, 1897)	MT994108	KROK055-19
Araneae	*Inermocoelotesinermis* (L. Koch, 1855)	MT994109	KROK056-19
Araneae	*Inermocoelotesinermis* (L. Koch, 1855)	OL874956	KROK132-20
Araneae	*Masosundevalli* (Westring, 1851)	MT994122	KROK074-19
Araneae	*Mermessustrilobatus* (Emerton, 1882)	MT994123	KROK075-19
Araneae	*Micronetaviaria* (Blackwall, 1841)	MT994124	KROK077-19
Araneae	*Micronetaviaria* (Blackwall, 1841)	OL874967	KROK145-20
Araneae	*Pardosaalacris* C.L. Koch, 1833	OL874968	KROK149-20
Araneae	*Pardosaalacris* C.L. Koch, 1833	MT994132	KROK085-19
Araneae	*Robertuslividus* (Blackwall, 1836)	MT994136	KROK089-19
Araneae	*Robertuslividus* (Blackwall, 1836)	OL874970	KROK153-20
Araneae	*Robertuslividus* (Blackwall, 1836)	OL874969	KROK154-20
Araneae	*Scotarguspilosus* Simon, 1913	MT994139	KROK078-19
Araneae	*Scotarguspilosus* Simon, 1913	OL874977	KROK146-20
Araneae	*Segestriasenoculata* (Linnaeus, 1758)	MT994140	KROK088-19
Araneae	*Tegenariasilvestris* L. Koch, 1872	MT994145	KROK057-19
Araneae	*Tegenariasilvestris* L. Koch, 1872	OL874981	KROK133-20
Araneae	*Tenuiphantesflavipes* (Blackwall, 1854)	MT994147	KROK079-19
Araneae	*Tenuiphantesflavipes* (Blackwall, 1854)	OL874982	KROK147-20
Araneae	*Tenuiphantestenebricola* (Wider, 1834)	MT994148	KROK082-19
Araneae	*Tenuiphantestenebricola* (Wider, 1834)	OL874983	KROK148-20
Araneae	*Trochosaterricola* Thorell, 1856	MT994150	KROK086-19
Araneae	*Trochosaterricola* Thorell, 1856	OL874984	KROK150-20
Araneae	*Walckenaeriaantica* (Wider, 1834)	MT994151	KROK083-19
Araneae	*Walckenaeriamitrata* (Menge, 1868)	MT994152	KROK084-19
Araneae	*Zoranemoralis* (Blackwall, 1861)	MT994153	KROK087-19
Araneae	*Zoranemoralis* (Blackwall, 1861)	OL874986	KROK151-20
Coleoptera	*Abaxovalis* (Duftschmid, 1812)	MT994068	KROK008-19
Coleoptera	*Abaxparallelepipedus* (Piller and Mitterpacher, 1783)	MT994069	KROK002-19
Coleoptera	*Carabuscatenulatus* Scopoli, 1763	MT994072	KROK019-19
Coleoptera	*Carabuscoriaceus* Linnaeus, 1758	MT994073	KROK006-19
Coleoptera	*Carabuscreutzeri* Fabricius, 1801	MT994074	KROK011-19
Coleoptera	*Carabuscroaticus* Dejean 1826	MT994075	KROK007-19
Coleoptera	*Carabusirregularis* Fabricius, 1792	MT994076	KROK020-19
Coleoptera	*Cychrusattenuatus* (Fabricius, 1792)	MT994087	KROK003-19
Coleoptera	*Dimaelateroides* Charpentier, 1825	MT994091	KROK023-19
Coleoptera	*Licinushoffmannseggii* (Panzer, 1803)	MT994111	KROK004-19
Coleoptera	*Molopspiceus* (Panzer, 1793)	MT994126	KROK017-19
Coleoptera	*Molopspiceus* (Panzer, 1793)	MT994125	KROK018-19
Coleoptera	*Molopspiceus* (Panzer, 1793)	MT994127	KROK012-19
Coleoptera	*Molopsstriolatus* (Fabricius, 1801)	MT994128	KROK015-19
Coleoptera	*Nebriadahlii* Sturm, 1815	MT994129	KROK021-19
Coleoptera	*Notiophilusbiguttatus* (Fabricius, 1779)	MT994131	KROK010-19
Coleoptera	*Platynusscrobiculatus* (Fabricius, 1801)	MT994133	KROK022-19
Coleoptera	*Pterostichusburmeisteri* Heer, 1837	MT994134	KROK005-19
Coleoptera	*Pterostichusoblongopunctatus* Fabricius, 1787	MT994135	KROK016-19
Coleoptera	*Stenichnuscollaris* (Müller, P.W.J. & Kunze, 1822)	MT994142	KROK014-19
Coleoptera	*Trechuscroaticus* Dejean, 1831	MT994149	KROK013-19
Geophilomorpha	*Clinopodescarinthiacus* (Latzel,1880)	MT994079	KROK025-19
Geophilomorpha	*Clinopodescarinthiacus* (Latzel,1880)	OL874927	KROK090-20
Geophilomorpha	*Clinopodescarinthiacus* (Latzel,1880)	OL874929	KROK098-20
Geophilomorpha	*Clinopodescarinthiacus* (Latzel,1880)	OL874928	KROK100-20
Geophilomorpha	*Dicellophiluscarniolensis* (C.L. Koch, 1847)	MT994089	KROK026-19
Geophilomorpha	*Dicellophiluscarniolensis* (C.L. Koch, 1847)	OL874945	KROK091-20
Geophilomorpha	*Dicellophiluscarniolensis* (C.L. Koch, 1847)	MT994090	KROK032-19
Geophilomorpha	*Eurygeophiluspinguis* (Brölemann, 1898)	MT994101	KROK027-19
Geophilomorpha	*Schendylaarmata* Brölemann, 1901	OL874972	KROK092-20
Geophilomorpha	*Schendylaarmata* Brölemann, 1901	OL874971	KROK102-20
Geophilomorpha	*Schendylatyrolensis* Meinert, 1870	MT994138	KROK029-19
Geophilomorpha	*Schendylatyrolensis* Meinert, 1870	OL874976	KROK095-20
Geophilomorpha	*Strigamiaacuminata* (Leach, 1814)	MT994143	KROK030-19
Geophilomorpha	*Strigamiaacuminata* (Leach, 1814)	OL874979	KROK096-20
Geophilomorpha	*Strigamiatranssilvanica* Verhoeff, 1928	MT994144	KROK031-19
Lithobiomorpha	*Eupolybothrusgrossipes* (C. L. Koch, 1847)	MT994099	KROK048-19
Lithobiomorpha	*Eupolybothrustridentinus* (Fanzago, 1874)	MT994100	KROK035-19
Lithobiomorpha	*Harpolithobiusgottscheensis* Verhoeff, 1937	MT994105	KROK036-19
Lithobiomorpha	*Harpolithobiusgottscheensis* Verhoeff, 1937	OL874951	KROK103-20
Lithobiomorpha	*Lithobiusanici* sp.n.	MT994141	KROK043-19
Lithobiomorpha	*Lithobiuscarinthiacus* Koren, 1992	MT994112	KROK044-19
Lithobiomorpha	*Lithobiuscastaneus* Newport, 1844	MT994113	KROK037-19
Lithobiomorpha	*Lithobiusdentatus* C.L.Koch, 1844	MT994116	KROK038-19
Lithobiomorpha	*Lithobiusdentatus* C.L.Koch, 1844	OL874961	KROK104-20
Lithobiomorpha	*Lithobiusdentatus* C.L.Koch, 1844	MT994115	KROK046-19
Lithobiomorpha	*Lithobiusforficatus* (Linnaeus, 1758)	MT994117	KROK047-19
Lithobiomorpha	*Lithobiuslatro* Meinert, 1872	OL874962	KROK105-20
Lithobiomorpha	*Lithobiuslatro* Meinert, 1872	MT994118	KROK039-19
Lithobiomorpha	*Lithobiuslatro* Meinert, 1872	OL874963	KROK109-20
Lithobiomorpha	*Lithobiuspelidnus* Haase, 1880	OL874964	KROK111-20
Lithobiomorpha	*Lithobiustenebrosus* Meinert, 1872	MT994120	KROK041-19
Lithobiomorpha	*Lithobiustenebrosus* Meinert, 1872	OL874965	KROK108-20
Lithobiomorpha	*Lithobiusvalidus* Meinert, 1872	MT994121	KROK042-19
Lithobiomorpha	*Lithobiusvalidus* Meinert, 1872	OL874966	KROK106-20
Scolopendromorpha	*Cryptopshortensis* Donovan, 1810	OL874934	KROK125-20
Scolopendromorpha	*Cryptopshortensis* Donovan, 1810	OL874933	KROK126-20
Scolopendromorpha	*Cryptopsparisi* Brölemann, 1920	OL874941	KROK119-20
Scolopendromorpha	*Cryptopsparisi* Brölemann, 1920	OL874940	KROK120-20
Scolopendromorpha	*Cryptopsparisi* Brölemann, 1920	MT994086	KROK050-19
Scolopendromorpha	*Cryptopsparisi* Brölemann, 1920	OL874939	KROK121-20
Scolopendromorpha	*Cryptopsparisi* Brölemann, 1920	OL874942	KROK122-20
Scolopendromorpha	*Cryptopsparisi* Brölemann, 1920	OL874943	KROK123-20
